# The Role of Gene Therapy in Premature Ovarian Insufficiency Management

**DOI:** 10.3390/biomedicines6040102

**Published:** 2018-11-01

**Authors:** Ihor Atabiekov, Elie Hobeika, Ujalla Sheikh, Abdeljabar El Andaloussi, Ayman Al-Hendy

**Affiliations:** Departments of Obstetrics and Gynecology, University of Illinois at Chicago, 820 South Wood Street, Chicago, IL 60612, USA; i.atabiekov@gmail.com (I.A.); Elieh@uic.edu (E.H.); ujallasheikh@gmail.com (U.S.)

**Keywords:** premature ovarian insufficiency, POI, gene therapy, menopause, *SAL-like 4* genes, SALL4, follicle-stimulating hormone (FSH), basonuclin-1, replication-incompetent adenoviral vector, Ad, stem cells, SC

## Abstract

Premature ovarian insufficiency (POI) is a highly prevalent disorder, characterized by the development of menopause before the age of 40. Most cases are idiopathic; however, in some women the cause of this condition (e.g.; anticancer treatment, genetic disorders, and enzymatic defects) could be identified. Although hormone-replacement therapy, the principal therapeutic approach for POI, helps alleviate the related symptoms, this does not effectively solve the issue of fertility. Assisted reproductive techniques also lack efficacy in these women. Thus, an effective approach to manage patients with POI is highly warranted. Several mechanisms associated with POI have been identified, including the lack of function of the follicle-stimulating hormone (FSH) receptor, alterations in apoptosis control, mutations in *Sal-like 4* genes, and thymulin or basonuclin-1 deficiency. The above mentioned may be good targets for gene therapy in order to correct defects leading to POI. The goal of this review is to summarize current experiences on POI studies that employed gene therapy, and to discuss possible future directions in this field.

## 1. Introduction

Premature ovarian insufficiency (POI) affects 1% of women by 40 years of age, and fewer than 0.01% of patients are younger than the age of 35 [1,2]. It is defined by the development of menopause before 40 years of age. Although the majority of cases remain idiopathic, a detailed history and physical examination, in addition to a workup, should be initiated to investigate identifiable etiologies [3]. The different etiologies of POI are described in Table 1. Follicle-stimulating hormone-receptor (FSHR) gene polymorphisms, chromosomal defects, and autoimmune and enzymatic disorders are among the known causes. Much effort has been made in order to identify genes responsible for POI development; today, the genetic cause of this condition is found in up to 25% of patients [4]. FMR1 [5], FIGLA [6], BMP15 [7], FSHR [8], FOXL2 [9], GDF9 [4], NOBOX [10], INHA [11], and STAG3 [12] are examples of gene mutations that are seen in some POI women.

It is important to also mention that the most recent report of the American Cancer Society for 2016–2017 has estimated that female cancer survivors of 49 years of age and younger are estimated to be over 1 million as of 1 January 2016. This emerging population significantly increases the prevalence of women with POI, and renders it a major healthcare problem that warrants therapy and management [13].

Clinical manifestations of POI, similar to menopause, are signs of hypoestrogenism due to ovarian dysfunction. Those include oligomenorrhea, with menopausal symptoms such as vaginal dryness, decreased libido, and vasomotor symptoms. However, the absence of these symptoms does not rule out diagnosis in the presence of appropriate laboratory findings. Since menstrual irregularities are the most common presenting manifestation of POI, other etiologies of secondary amenorrhea should be excluded (Table 2).

Early onset hypoestrogenism has deleterious effects on a woman’s general health and wellbeing. In addition to symptoms affecting the woman’s quality of life, such as vasomotor symptoms and vaginal atrophy, and accelerated bone loss leading to osteoporosis, increased cardiovascular morbidity and mortality are the main consequences of estrogen deficiency, especially when it occurs in women of reproductive age. Recent reports also suggest a role of estrogen in mental health, with increased rates of dementia and neurological disorders such as Parkinson’s disease in patients with early-onset menopause [14,15,16]. Interestingly, spontaneous ovulation can still occur in women with POI with ovaries in situ [17]. Patients should be counseled that the chances of spontaneous conception, although low for age-matched controls, are still present, and can reach a lifetime probability of 10% [18].

Hormone-replacement therapy (HRT) is a mainstay in the treatment of POI from the time of diagnosis until at least the average age of menopause. Contemporary studies and reports do not fully support its use beyond that age. HRT helps counteract the effects of hypoestrogenism by promoting bone and cardiovascular health, in addition to improving quality of life through the resolution of vasomotor symptoms and vaginal atrophy. In terms of reproductive outcomes, HRT may improve spontaneous ovulation rates, but it is not the standard of care for women desiring conception. Assisted reproductive technologies are seldom fruitful due to a diminished ovarian reserve in addition to poor ovarian response. Adoption or oocyte donation are commonly recommended for patients who haven’t completed child bearing at time of diagnosis [19]. In women with newly diagnosed cancer, in vitro fertilization with oocyte and embryo banking is an option prior to initiation of medical or radiation cytotoxic therapy and the induction of POI [20,21]. Cryopreservation of fresh ovarian tissue prior to cytotoxic treatment with subsequent transplantation has not yet been fully implemented, but results of pilot studies have been promising [22]. Nevertheless, the concern of simultaneous cryopreservation of carcinogenic cells in the ovarian cortex, mainly in the case of lymphomas and leukemia, leading to cancer recurrence after autologous transplantation has always been major with this technique.

The data on effectiveness and teratogenicity of other agents for POI patients (bisphosphonates, raloxifene, strontium ranelate, herbal remedies) are currently lacking [23].

Thereby, effective treatment of POI is needed, especially to restore reproductive function. Gene therapy is a potentially promising avenue that has recently been attracting interest. The goal of this review is to summarize the available information on gene-therapy attempts in POI, and to underline the potential target genes that may be influenced in future studies.

## 2. Gene Therapy for FSH-Receptor Defect Correction

Females are born with approximately one million primordial follicles, arrested at the prophase of the first meiotic division. The majority undergo atresia, and only 600,000 are present at puberty. When a girl is born, all follicles are arrested at the early stage of development; this changes during puberty, when stimulation of the ovaries by the FSH occurs. These data were generated using a transgenic mouse with a sertoli tumor [24]. 

FSH signaling through its receptor (FSHR) is essential for this process, as well as for spermatogenesis in adult males [25]. Folliculogenesis is a lengthy process, involving the growth and maturation of the follicle from the primordial to the preovulatory stage. Approximately 400 follicles of the total ovarian follicle pool ovulate during the reproductive years of a woman. 

Aittomaki et al. identified a point mutation (C566T) in the *FSHR* gene and showed a substantial decrease of FSH/FSHR binding and, thus, a failure to increase intracellular cAMP levels in mutated FSHR transfection experiments [24]. Males with C566T mutation of both alleles had decreased fertility, whereas homozygous females had POI due to resistant-ovary syndrome (ROS) [25]. Usually, this is seen as primary amenorrhea with high-serum FSH, somewhat decreased secondary sex features, and normal karyotype and genitalia [24]. Similar signs are seen in other mutations of the *FSHR* gene [8,26,27,28]. The follicles in these women do not develop, and continuous atresia occurs [24]. There is still no effective therapy for these conditions, and chances of spontaneous pregnancy are very low. FSH stimulation of the ovaries is ineffective [29]. The only method that allows pregnancy is in vitro fertilization (IVF) using donated eggs, which is very expensive, ethically unacceptable for many women, and results in a genetically unrelated fetus. 

One of the most commonly used types of vector for gene therapy are replication-incompetent adenoviruses (Ad) that have been proven to be safe [30]. Al-Hendy et al. used Ad to transfect both human and Eker rat uterine fibroid cells (ELT3) with a dominant negative estrogen receptor (ER) to inhibit the estrogen pathway, and observed shrinkage of the size of leiomyoma [31,32,33]. No safety issues have been observed. Ghadami et al. developed an Ad vector carrying a *full-length human FSHR* (*hFSHR*) gene (*Ad-hFSHR*), and demonstrated its ability to restore FSH activity in C566T-mutated cells [34].

Interestingly, when follitropin receptor knockout mice (FORKO), a good model of hypergonadotropic hypogonadism with infertility and hypoplastic internal genitalia secondary to a deleted *FSHR* gene and resembling human ROS, are bilaterally injected with Ad-hFSH into the ovaries, demonstrated folliculogenesis, a two–threefold rise in estrogens, serum FSH reduction, and body- and genitalia-weight increase. In addition, the ovaries of these animals started to show FSHR expression [35,36,37].

The intraovarian injection of Ad-LacZ did not show systemic viral spread or fertility disturbances in mice, corroborating previous observations. Viral genes were not detected in mouse pups, injected into the ovaries with Ad-LacZ; thus, germ line transmission was also excluded.

Unfortunately, ovulation or pregnancy was not achieved in injected mice after 12 weeks of observation. This did not also occur after injection of both the study and control groups with PMSG followed by hCG. Mice were mated with normal males, but no pregnancies were observed. This may be explained by the use of a strong dominant CMV5 promoter in the Ad-hFSHR vector, not allowing the downregulation of FSHR in the later stages of follicular development (luteal phase). This may be fixed with the rebuilt Ad-hFSHR using an authentic human promoter. The data from this study show that Ad-hFSHR injection into the ovaries of FORKO mice led to partial hormonal correction and the mobilization of follicles up to the antral phase with subsequent arrest, thus not reaching ovulation [35]. This is a promising direction of future investigations in the area of POI, related to defective ovarian FSH action, where gene therapy may play an important role.

## 3. Sal-Like 4 Genes as a Target of Gene Therapy in POI

*Sal-like 4* (*SALL4*) genes are highly expressed in vertebrates’ embryonic and adult stem cells, giving rise to their stemness. Postpartum, they are only found in adult stem/stemlike cells of bone-marrow and gonadal origin [38,39,40]. As these genes are involved in cell growth and development, they may be a reasonable target for gene therapy. It is worth mentioning that some disorders, including POI, may be seen in patients with a mutated *Sall4* gene [38]. Sequence screening for Chinese patients with SALL4-related syndromes (ventricular septal defects and POI) identified several distinct variants of *SALL4* genes [41,42]. The chromosomal locus of human SALL4 is 20q13.13-q13.2, while for mice it is chromosome 2H3 [39,40]. Aguila et al. have shown that SALL4 can facilitate the regeneration of bone marrow and the division of hematopoetic stem cells (HSCs) in vitro and in vivo [43]. Interestingly, C.541G>A (p.Val181Met) and c.2449A>G (p.Thr817Ala) SALL4 mutations were found in 100 women with POI compared to 300 healthy controls [41]. A thorough look into the SALL4 pathway and the study of its client proteins may clear up the appropriate approach for the stem cell-based treatment of many disorders, including POI, in the future. However, caution must be used, as SALL4 is known to function as an oncogene in various germ cell-related tumors [44,45,46,47] and neoplasms of gastrointestinal origin [48,49,50,51,52]. The SALL4-related cell stemness concept, for example, is used in SALL4–HSC transplantation. Nevertheless, the data on the connections between SALL4 functions and the possibility of their clinical use are still lacking.

## 4. Programmed Cell Death and Sphingomyelinase Gene

According to the recent data, apoptosis is considered to be the leading mechanism of oocyte loss, both developmental and secondary to malignancy treatment [53]. This information may provide us with new tools to delay menopause, reducing apoptosis-related follicular atresia, or protect ovarian function during anticancer treatment [54,55]. Apoptotic pathway is complex and it includes multiple steps that can potentially be targeted by therapeutic intervention [56]. However, several studies reported that targeting the final apoptotic stages (e.g.; inhibiting caspases) leads to a switch from apoptotic pathway in cells destined for death to a process similar to primary necrosis, putting the potential benefit of such measures under doubt [57,58,59]. Thus, inhibiting the earlier steps of programmed cell death may theoretically be more effective in cell preservation.

One of such potential targets is ceramide, a secondary messenger involved in proapoptotic signaling [60]. The multistep process of ceramide utilization is partially regulated by sphingosine kinase and finally results in sphingosine-1-phosphate (SP) production, which counteracts ceramide, blocking apoptosis progression [61,62,63]. Morita et al. (2000) studied the sphingomyelin pathway in ovaries with the goal to develop a new approach of early-step apoptosis control in prospect [64]. Sphingomyelin phosphodiesterase 1 (SPD1) is a crucial enzyme needed for programmed death initiation, as it hydrolyzes sphingomyelin, thus unblocking ceramide signaling. The neonatal female mice deficient in SPD1 (SPD1^−/−^) had significantly higher content of primordial follicles per ovary compared to the wild-type controls; primary and small preantral follicle hyperplasia, and greater egg reserve, were also observed in SPD1^−/−^ mice. Moreover, explainable symptoms resembling the human Niemann–Pick syndrome were observed in the murine study group in postnatal life [65]. When fetal ovaries from both groups of mice were collected for in vitro culture, wild-type fetal ovaries demonstrated time-dependent apoptosis initiation in germ cells, whereas in SPD1^−/−^ mice it was significantly retarded, serving as a logical explanation for the larger egg pool in newborn mutant mice [64].

It’s worth mentioning that sphingomyelin degradation is crucial for apoptosis initiation rather than de novo ceramide synthesis, which was been proved in experiments with fumonisin-B1, a selective blocker of ceramide synthase [66,67]. Morita et al. (2000) also observed similar results in fetal ovarian culture morphology in both SPD1-deficient and nondeficient but treated with SP samples [64]. When isolated oocytes from SPD^−/−^ and wild-type mice were cultured in the presence of doxorubicin (anticancer drug), the study group demonstrated resistance to it, whereas the control group showed robust apoptosis [64,68]. Several studies showed that the protective action of SP on neurons and oocytes was not dependent on GI-coupled endothelial differentiation and growth receptors [69,70,71]. SP also has a radioprotective action on gonads, as its administration prior to radiation therapy in mice showed dose-dependent preservation of the follicular reserve, whereas almost complete loss of primordial follicles was observed in control group. Observation for two weeks after treatment proved that the follicles in SP-treated mice were totally functional and viable [64].

## 5. Neonatal Thymulin Gene Therapy

Congenitally athymic (nude) mice have pituitary-gonadal axis developmental abnormalities resulting in delayed sexual maturation, decreased fertility, and a shorter reproductive period, related to accelerated ovarian follicular atresia with subsequent POI [72,73,74,75]. When neonatal mice undergo thymectomy, similar features occur [76,77]. Thymulin is known to demonstrate gonadotropin-releasing action, regulate sexual development in females, and modulate gonadotropin-dependent steroidogenesis in the gonads [78,79,80,81,82]. 

Reggiani et al. (2012) showed that the immunoneutralization of thymulin in normal mice postnatally results in lower levels of gonadotropins at puberty. They also used neonatal thymulin gene therapy (NTGT) in nude mice, which resulted in thymulin production and release into the bloodstream in these animals, and prevented gonadotropin deficiency, which typically occurs in nude mice [83,84]. NTGT used a recombinant adenovirus (RAd) carrying the *methionine-FTS* (5′-ATGCAGGCCAAGTCGCAGGGGGGGTCG-AACTAGTAG-3′) gene (*metFTS*). RAd-green fluorescent protein (GFP) was used for the control group. Both groups were injected with a corresponding RAd on Day 1 after birth. Nude mice were tested on Day 70, and active circulating serum thymulin was increased only in the study group of both homo- and heterozygous nude mice. The levels of thymulin were only slightly lower in RAd-FTS nu/nu mice compared to nu/+ controls, and RAd-FTS heterozygotes had comparable thymulin levels with the controls. NTGT was able to prevent gonadotropin-releasing hormone (GnRH) neuronal deficiency (anterior hypothalamus and preoptic nucleus), typical for nude mice. Nu/+ mice had normal gonads with follicles in all stages of development and normal corpora lutea, whereas nu/nu controls had anomalous ovaries with low follicle count, no preovulatory follicles, and high amount of atretic follicles compared to normal controls. Treated nu/nu showed an ovarian picture comparable to nu/+ controls. In addition, nu/nu that received NTGT had normal serum estrogens versus untreated nu/nu. NTGT was also able to attenuate the vaginal opening delay observed in nude mice [83].

## 6. Basonuclin-1 Deficit as a Cause of POI

Huang et al. (2018) identified another causative gene responsible for POI development in some of the patients [85]. BNC1 is located on Chromosome 15 and is known to be expressed in oocytes. Its defect produces truncated protein, leading to haploinsufficiency or a gain of abnormal functions. This abnormal basonuclin-1 causes reduced meiosis in oocytes. 

Using whole-exome sequencing, the above-mentioned group of scientists identified a 5 bp deletion in the *BNC1* gene encoding basonuclin-1. This frameshift mutation has an autosomal dominant type of inheritance and causes POI, running in families. In vivo experiments support these data: Bnc1^tr/tr^ mice did not produce pups after mating with wild-type males, and Bnc1^tr/+^ group were subfertile, whereas Bnc1^+/+^ mice (control group) showed normal fertility. The hormonal level of the latter three groups of animals showed no difference at eight weeks; however, at 36 weeks, significantly lower estrogen levels and higher FSH and LH in the Bnc1^tr/tr^ and Bnc1^tr/+^ groups were detected compared to controls. Expectedly, Bnc1 mutant mice showed smaller ovaries with a lower follicular count [85].

## 7. Future Directions

The Ad vector and its delivery were safe and well tolerated by mice. Still, as was said earlier, pregnancy did not occur in the treated animals, probably due to the dominance of the Ad-hFSHR vector promoter, leading to a lack of FSHR downregulation in the further stages of follicular development and, thus, arrest at the antral stage. Future work is likely to be done on the modification of this vector in order to unblock the later stages of follicular development and reach ovulation.

Distinct mutations of *SALL4* genes associated with POI development are theoretically good targets for gene therapy. Not much is known about the SALL4 functioning and signaling pathways, which need to be studied thoroughly. In addition, caution should be used, as SALL4 are powerful proto-oncogenes and are known to play a role in various tumors.

Targeting genes responsible for apoptosis may potentially prevent POI, as it is known that programmed cell death is a leading mechanism of follicular atresia. Undoubtedly, this must be very specific and well controlled, as apoptosis is the major process of every cell’s functioning, and its defect may lead to a variety of pathologic conditions, including neoplasia.

Other genes known to be involved in POI development may also be targeted for gene therapy. However, further investigation of their functioning and more ways of correcting their defects are needed.

**Figure 1:**
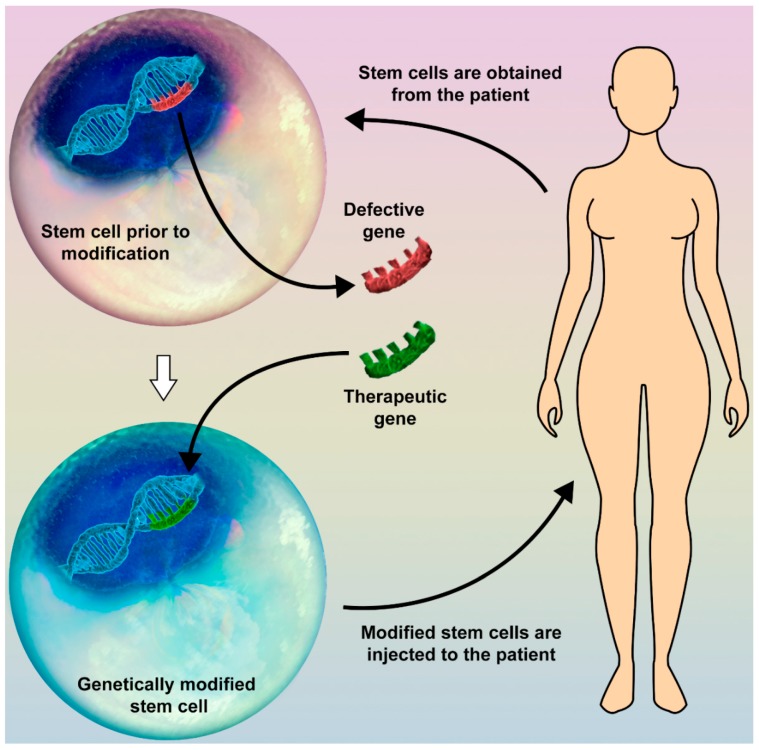
Gene therapy in combination with the use of stem cells (SC) may open up a new field in regenerative medicine. A possible way to modify human genes without using a viral vector is SC therapy. Different SC types may be obtained from various types of tissue of the human body. The target gene is replaced with the therapeutic one, and the modified SC is then injected back into the patient’s body (e.g., peripheral blood, target organ). These altered SC may produce a colony of new specific cells or influence the function of the surrounding tissue.

We believe that much effort should be made in the future in the field of stem-cell (SC) therapy. Since they have multiple mechanisms of affecting types of tissue, including paracrine regulation of cell functioning, stimulation of cellular growth and division, and the ability to differentiate into target cells, SC greatly attract the interest of many researchers all over the world. Theoretically, target genes of SC may be altered in vitro and reinjected, thus giving the opportunity to avoid the use of a viral vector (Figure 1). The resultant SC may potentially give rise to a new, modified pool of cells, functioning in the desired way. This would open up an exciting new field in regenerative medicine. Unfortunately, as of now the available data are deficient, thus motivating us for new investigations.

## Figures and Tables

**Table 1:** Causes of premature ovarian insufficiency.

Idiopathic	
**Primary**	Chromosomal disease
	Follicle-stimulating hormone-receptor (FSHR) gene polymorphism
	Mutation of inhibin B
	Autoimmune disorders
	Enzymatic defects
**Secondary**	Cancer treatment (chemotherapy, radiotherapy)
	Ovarian surgery
	Uterine artery embolization
	Infections (mumps etc.)

**Table 2:** Other causes of secondary amenorrhea that should be excluded.

**Physiologic**	Pregnancy
**Intrauterine adhesions**	Asherman syndrome
	Tuberculous endometritis
**Hypothalamic**	Functional hypothalamic amenorrhea
**Pituitary**	Prolactinoma
	Empty sella syndrome
	Sheehan syndrome
	Cushing syndrome
**Ovarian**	Polycystic ovarian syndrome
**Others**	Hypothyroidism
	Ovarian tumors
	Congenital adrenal hyperplasia
	Adrenal tumors
